# Modified Quad surgery significantly improves the median nerve conduction and functional outcomes in obstetric brachial plexus nerve injury

**DOI:** 10.1186/1750-1164-7-5

**Published:** 2013-05-28

**Authors:** Rahul K Nath, Nirupuma Kumar, Chandra Somasundaram

**Affiliations:** 1 Texas Nerve and Paralysis Institute, 6400 Fannin st, Houston, TX 77030, USA

**Keywords:** Obstetric brachial plexus nerve injury, Electromyography, Nerve conduction study, Somatosensory evoked potentials, Shoulder abduction, Modified Quad surgery

## Abstract

**Background:**

Nerve conduction studies or somatosensory evoked potentials (SSEPs) have become an important tool in the investigation of peripheral nerve lesions, and is sensitive in detecting brachial plexus nerve injury, and other nerve injuries.

To investigate whether the modified Quad surgical procedure improves nerve conductivity and functional outcomes in obstetric brachial plexus nerve injury (OBPI) patients.

**Methods:**

All nerves were tested with direct functional electrical stimulation. A Prass probe was used to stimulate the nerves, and recording the response, the compound motor action potential (CMAP) in the muscle. SSEP monitoring was performed pre- and post modified Quad surgery, stimulating the median and ulnar nerves at the wrist, the radial nerve over the dorsum of the hand, recording the peripheral, cervical and cortical responses. All patients have had the modified Quad surgery (n = 19). The modified Quad surgery is a muscle release and transfer surgery with nerve decompressions. All patients were assessed preoperatively and postoperatively by evaluating video recordings of standardized movements, the modified Mallet scale to index active shoulder movements.

**Results:**

The cervical responses were significantly lower in amplitude in the affected arm than the un-affected arm. The median nerve conduction was significantly improved from 8.04 to 9.26 (P < 0.022) post-operatively. The shoulder abduction was also significantly improved (pre-op 30° ± 23.3 to 143° ± 33.7, p < 0.0001), with a mean follow-up of 43 months after the modified Quad surgery in these patients.

**Conclusion:**

Median nerve conduction, and shoulder abduction were significantly improved in OBPI children, who have undergone the modified Quad procedure with neuroplasty, internal microneurolysis and tetanic stimulation of the median nerve.

## Background

Indications and timing, as well as type of surgical repair are important questions in the management of obstetric brachial plexus injury (OBPI). Delay to time of surgery often results in progressive worsening of deformity in the shoulder joint as contractures progress quickly over time in a rapidly growing infant [1]. Diagnostic tools used to identify which lesions are permanent in OBPI include CT, MRI, myelogram, and electromyography (EMG) as well as nerve conduction velocity (NCV) studies [2-5]. Distinguishing preganglionic (avulsion) from postganglionic (rupture) lesions is critical, and can be difficult at initial presentation based on clinical examination alone in these infants [6,7].

Nerve conduction studies or somatosensory evoked potentials (SSEPs) have become an important tool in the investigation of peripheral nerve lesions [2], and are sensitive in detecting brachial plexus nerve injury [3-8], and other nerve injuries [9]. The median nerve (MN)-SSEPs, notably the subcortical tracings have been demonstrated to be more useful for the detection of some neurological disorders in children than posterior tibial nerve (PTN)-SSEPs [10]. Furthermore, a significantly lower nerve conduction velocity was found in the median nerve of the injured arm of anterior interosseous nerve syndrome patients, compared to the normal arm [11].

The modified Quad procedure was performed on appropriate patients at our institute [12]. Nerve conduction testing was done just before the modified Quad surgery in these patients, and these values were compared statistically with SSEPs monitored in the same patients later in time prior to indicated bony surgery, the triangle tilt. We found there was a significant improvement in median nerve (MN) conduction, and shoulder abduction after the modified Quad surgery in this patient population.

## Methods

A retrospective chart review was conducted based on surgical cases performed at the Texas Nerve and Paralysis Institute. Inclusion criteria included infants, who showed lack of antigravity biceps functions pre-operatively, and had undergone the modified Quad surgery between 2004 and 2010. Nineteen OBPI patients, aged between 4 and 8 months (average 5 ± 0.1) were found who fit these criteria with a mean follow-up time of 43 months. This was a retrospective study of patient charts, which exempted it from the need for IRB approval in the United States. Patients were treated ethically in compliance with the Helsinki declaration. Documented informed consent was obtained for all patients.

The nerve involvement was C5, 6 (N = 1), C5, 6, 7 (N = 4) and C5, 6, 7, 8 (N = 5), and with T1 involvement (N = 9).

SSEP monitoring was performed pre- and post-modified Quad surgery, stimulating the median and ulnar nerves at the wrist, the radial nerve over the dorsum of the hand, and recording the peripheral, cervical and cortical responses. All patients have had the modified Quad surgery (n = 19). All surgical procedures were performed by the lead author. The modified Quad [12] is a modification of the combination of muscles released and their insert positions to improve upon a previously described operation [13]. In the modified Quad procedure, the latissimus dorsi, teres major, subscapularis, and pectoralis muscle contractures are released [12]. Additionally, the axillary nerve is neurolysed. Empirically it was thought that the median nerve may be compressed by the pectoralis major muscle contracture as the two structures meet in the area of the anterior axillary fold.

### Mallet grading

All patients were assessed preoperatively and postoperatively by evaluating video recordings of standardized movements using the modified Mallet scale to index active shoulder movements [14].

### Statistical analysis

Paired Student’s t-tests were conducted using Microsoft Excel 2003 with the Analyze-It plug-in (Redmond, WA; and Leeds, UK) to determine if differences between preoperative and postoperative shoulder abduction, and SSEPS for each function were statistically significant. The p values were two-tailed and considered significant if less than or equal to 0.05.

## Results

**Figure 1:**
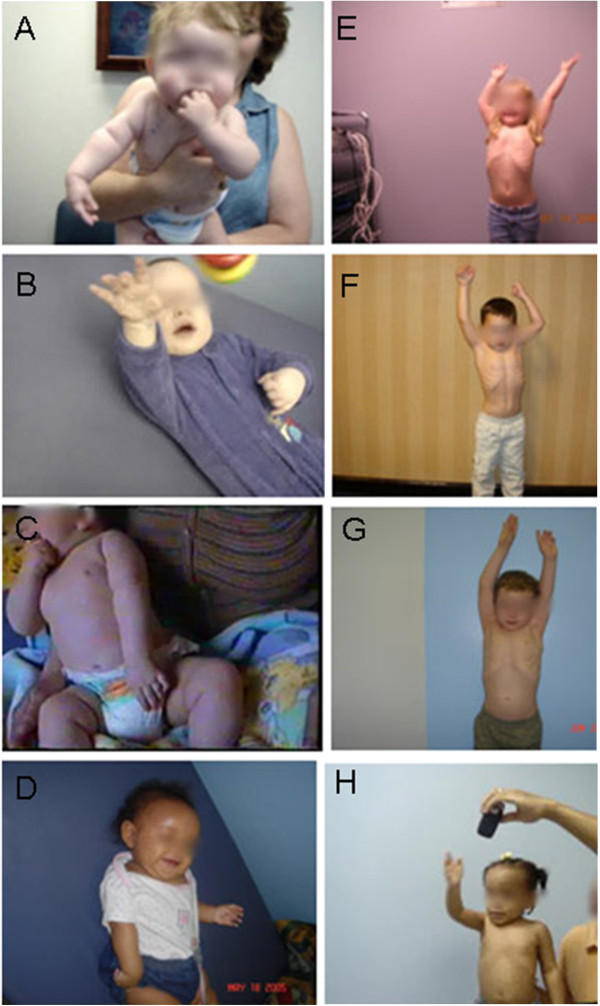
**Comparisons of pre- and post-operative shoulder abduction in OBPI patients. A**) Preoperative photograph of a 6 month old girl (**A**), 7 month old boys (**B**), (**C**) and (**D**) demonstrating limitation of shoulder movement; same patients 2.3 years (range 1.5 to 3 years) after modified Quad surgery with almost normal shoulder abduction (**E**, **F**, **G** and **H**).

The cervical responses were significantly lower in amplitude in the affected arm than the un-affected arm. The median nerve conduction was significantly improved from 8.04 to 9.26 (P < 0.022) post-operatively (Table 1). We were also able to find significant functional improvement in these patients, who have undergone the modified Quad procedure. The postoperative abduction was significantly improved from 30° ± 23.3 to 143° ± 33.7 (p < 0.0001) in these OBPI patients (Table 2 and Figure 1).

**Table 1:** SSEPs (CERV) before and after modified Quad surgery in OBPI patients

		**Ulnar-Lat Mean ± SEM**	**Ulnar-Amp Mean ± SEM**	**Median-Lat Mean ± SEM**	**Median-Amp Mean ± SEM**
Pre- mod Quad	Affected	8.04 ±3.84	2.51 ±1.98	8.04 ±3.84	1.91 ±1.49
Post- mod Quad	Affected	8.6 ± 3.16	2.44 ± 1.63	9.26 ±2.98	2.32 ±1.57
N = 19	P=	0.50	0.54	**0.022**	0.12

Median nerve conduction improved significantly after MQ surgery in these patients.

**Table 2:** Abduction before and after modified Quad surgery in OBPI patients

**Function**	**Pre-op**	**Post-op**	**Significance**
	**Mean ± SEM**	**Mean ± SEM**	**p < 0.05**
Abduction (^o^)	30° ± 23.3	143° ± 33.7	p < 0.0001
			(n = 19)

## Discussion

Vredeveld et al., 2000 [5], and Colon et al., 2003 [4] reported the existence of an extensive innervation by the somatosensory system in infants with obstetric upper brachial palsy. C5 stimulation shows abduction of the limb and some external rotation, while C6 stimulation shows elbow flexion against gravity with some supination [6].

Unlike nerve grafting, neuroplasty with microneurolysis and tetanic stimulation has an immediate effect on muscle recovery [15]. Thus, such procedures on the axillary nerve effectively improves shoulder function since they may help to slow the acquisition of the deformity, and contractures due to progressive muscle imbalance that occur with growth. Combined with modified Quad surgery axillary neuroplasty can have a significant impact on the underlying pathophysiology of the overall shoulder joint dysfunction caused by OBPI. This was the basis for testing the distal branches of the brachial plexus specifically the median nerve, as significant improvements in hand function such as finger and wrist flexion were routinely noted immediately following modified Quad surgery.

The significant improvement noted in median nerve conduction when compared to the ulnar nerve, probably occurs because the median nerve gets decompressed during the modified Quad surgery. The median nerve decompression probably occurs due to the pectoralis major muscle contracture release during this surgery.

Performed alone neurolysis does not address the pathophysiology of OBPI injuries and does not provide relief from contractures and improve the overall stability of the shoulder joint [16-19]. By combining our approach of neurolysis with stimulation, and contracture release, we were able to achieve significant long-term functional improvements in these patients, in their shoulder function as well as hand function through improved median nerve function.

## Conclusion

Median nerve conduction, and shoulder abduction were significantly improved in the current series of OBPI patients, who have undergone the modified Quad procedure.

## Competing interests

The authors declare that they have no competing interests.

## Authors’ contributions

RKN conceived the study, performed the surgeries, participated in the design of the study, help to write and revised the manuscript. NK gathered and analyzed the data, and helped to draft the manuscript. CS participated in the design of the study, gathered and analyzed the data, drafted and revised the manuscript. All authors have read and approved the final manuscript.
